# Action of silicon on the activity of antioxidant enzymes and on physiological mechanisms mitigates water deficit in sugarcane and energy cane plants

**DOI:** 10.1038/s41598-022-21680-9

**Published:** 2022-10-19

**Authors:** Gelza Carliane Marques Teixeira, Renato Mello de Prado, Antonio Márcio Souza Rocha, Antonio Santana Batista de Oliveira Filho, Gilmar Silveira da Sousa Junior, Priscila Lupino Gratão

**Affiliations:** 1 Laboratory of Plant Nutrition, Department of Soils and Fertilizers, São Paulo State University (UNESP), Jaboticabal, São Paulo, Brazil; 2 Laboratory of Biogeochemistry, Department of Technology, São Paulo State University (UNESP), Jaboticabal, São Paulo, Brazil; 3 Laboratory of Plant Physiology, Department of Biology Applied to Agriculture, São Paulo State University (UNESP), Jaboticabal, São Paulo, Brazil

**Keywords:** Plant sciences, Plant physiology, Plant stress responses

## Abstract

Production of sugarcane and more recently of energy cane strengthen renewable bioenergy production capacity. However, droughts resulting from climate change have limited the production of these crops. One of the strategies to attenuate water deficit damage in these crops is the use of silicate, which contributes to plant physiology. This strategy is likely to increase water use efficiency, thus promoting crop sustainability. Notwithstanding, studies on this issue are still incipient. This study assesses whether Si applied via fertigation and foliar spraying in the seedling production phase and as a complement after seedling transplanting to the soil is efficient in attenuating water deficit in sugarcane and energy cane. The study further elucidates physiological and biochemical mechanisms involved in this process. For this, the authors conducted two experiments: one with sugarcane and the other with energy cane. Treatments were arranged in randomized blocks with 5 replications, in a 2 × 2 factorial scheme. Factors consisted of the absence (-Si) and presence of Si (+ Si) applied via fertigation and foliar spraying; and two water regimes: 70% (without water deficit) and 30% (severe water deficit) of the soil water retention capacity. Silicon was supplied during the formation phase of presprouted seedlings and during the transplanting of seedlings to pots filled with samples of Entisol (Quartzipsamment). In these pots, water regimes were induced from 7 to 160 days after transplanting. Severe water deficit reduced the water content and water potential of plants. This situation induced oxidative stress and impaired gas exchange and photosynthetic water use efficiency, reducing plant growth. Silicon supply via fertigation in association with foliar spraying in the seedling formation phase with complementation after transplanting was efficient in increasing Si accumulation in the plants. Silicon was effective in attenuating severe water deficit damage up to initial culm formation through mechanisms that maintain water and physiological balance by favoring the antioxidant defense system in sugarcane and energy cane plants.

## Introduction

Drought is one of the factors that most limit agricultural production^1^. It is noteworthy that drought conditions have worsened recently and are likely to increase due to climate change^2^, reducing sugarcane yield. This effect is important because the sugarcane growing area has expanded and currently occupies more than 100 countries^3^. This is due to sugarcane being a source of sustainable bioenergy that helps to reduce greenhouse gas emissions^4^. Irrigated sugarcane cultivation has expanded in this context, especially in top producing countries such as Brazil^5^, China^6^, and India^7^. Therefore, reducing water consumption and increasing water use economy are of great importance to ensure adequate resources and sustainable agricultural production^8^.

A strategy to reduce water deficit damage in crops is the use of silicon (Si). Silicon is the second most abundant element in soil, behind only oxygen. Silicon dioxide (SiO_2_) comprises 50–70% of the soil mass^9^. In the soil solution, however, available Si is in the form of monosilicic acid (H_4_SiO_4_) at low concentration (~ 1 mmol L^−1^)^10^. Silicon is a beneficial element absorbed in the form of H_4_SiO_4_. This form remains the same in the xylem sap, reaching the leaves through the transpiration gradient^11^. It concentrates in leaf tissue in polymerized form as amorphous and biogenic silica, constituting 90% of the absorbed Si and being contained in Si-cellulose structures present in the cell wall^12^.

The beneficial effects of Si on plants under water deficit prevent leaf water imbalance. This is because deposits of this element on cell walls reduce water loss by transpiration^13^. Silicon also induces an osmoregulatory response by modifying proline content that adjusts root hydraulic conductivity^14^. Moreover, it stimulates the activity of enzymes such as aquaporin^15^, increasing water absorption. The role of Si in regulating enzymatic^16^ and nonenzymatic^17^ antioxidant defense systems favor plant tolerance to drought. By decreasing oxidative stress, Si preserves and increases photosynthetic pigments^18^ while maintaining high photochemical efficiency^19^. This should increase the duration of active green leaves and consequently photosynthetic rate and plant growth. However, further studies are necessary to prove this hypothesis.

A high uptake of silicon is important for obtaining a biological benefit to the plant. This concerns Si application mode, which correlates with its source that can enhance biological effects and plant growth. Silicon can be applied to the soil in solid form using sources of limited solubility from calcium silicate. In addition, the element can be supplied either in fluid form via foliar spray or to the soil via fertigation using sources with high water solubility. These ways of applying Si have some important implications.

Silicon can be applied with the use of calcium silicate; however, it needs to be incorporated into the soil at high doses (1000 kg ha^−1^ of Si) that favor its dissolution^20^. Another way would be the use of high solubility sources (potassium silicate or sodium silicate). Soluble sources allow use via fertigation, which could reduce Si polymerization rates in the soil. This type of application uses low Si doses per area; however, these doses guarantee its adequate absorption by the plant. This is because fertigation allows the use of a solution with concentrations below 3 mmol L^−1^ Si, avoiding polymerization^21^. Previous studies have shown the efficiency of Si fertigation in optimizing the absorption of this element in forage plants^22^ and in presprouted sugarcane seedlings^17^. Notwithstanding, further studies on plant cane are needed, especially regarding stages with high relative growth rates. These stages usually occur between 84 and 135 days after transplanting^7^ depending on genotype and growing conditions^23^.

Studies addressing silicon focus on sugarcane and discuss physiological parameters only, without demonstrating additive effects of biochemical mechanisms. To our knowledge there are no reports on energy cane plants. However, it is noteworthy that water deficit induces oxidative stress, which increases the production of reactive oxygen species (ROS)^24^. Moreover, studies show increased planting of sugarcane and energy cane in areas with greater occurrence of water deficit that require a greater need for supplementary irrigation^5^. This strengthens the importance of using Si to promote rational use of irrigation water, which is a finite resource^25^.

Research has not yet described the effects of Si application via fertigation in association with foliar spraying during the production of presprouted seedlings with complementation after transplanting to the soil. The same can be said for the effects of Si application on the initial culm formation of sugarcane and energy cane.

However, it is important to evaluate two strategies involving Si optimization in crops. One would be the use of this element in its soluble form via fertigation as mentioned above. The other would be the use of Si after the formation of presprouted seedlings as well as after seedling transplanting to the soil. These strategies could maximize the biological benefits of this element in the plant to mitigate severe water deficit that normally occurs in the field for sugarcane and energy cane. Nevertheless, further studies on this topic are needed.

Therefore it is important to verify three hypotheses. The first hypothesis is: i) Si fertigation in association with foliar spraying in the seedling formation phase with complementation after transplanting (< 20 kg ha^−1^ Si) provides high accumulation of this element in the plants. The second hypothesis is: ii) Si promotes benefits by inducing enzymatic—catalase (CAT), superoxidase dismutase (SOD), and ascorbate peroxidase (APX)—and nonenzymatic (phenol and proline) antioxidant defense mechanisms, reducing oxidative stress. This would favor physiological aspects including water content and water use efficiency, pigments, photosynthesis, and consequently tillering, number of green leaves, and dry matter production of the two species under study. Finally, the third hypothesis is: iii) Si is efficient in attenuating severe water deficit damage up to the initial culm formation of sugarcane and energy cane.

To test these hypotheses, the authors of the present study conducted two experiments, one with sugarcane and the other with energy cane. The aim was to evaluate whether Si applied via fertigation in association with foliar spraying in the seedling production phase with complementation after transplanting to the soil is efficient in attenuating water deficit in sugarcane and energy cane. The authors also elucidated the physiological and biochemical mechanisms involved.

Acceptance of the hypotheses of this study enables understanding the synergistic mechanisms between silicon and the physiological and biochemical aspects of sugarcane and energy cane. This allows using the benefits of mineral nutrition with Si in plants to increase water use efficiency. Environmental and agronomic gains could thus be achieved by favoring sustainable establishment of these species even in drought regions.

## Material and methods

### Plant material and growing conditions

The authors conducted two experiments simultaneously in a greenhouse at the State University of São Paulo—UNESP from January to September 2019. Temperature and relative humidity inside the greenhouse were recorded daily with the aid of a thermohygrometer (U23-001, Sigma Sensors, Brazil) (S1).

Presprouted seedlings of two species were used: *Saccharum* Spp. (sugarcane—variety RB 966,928—experiment I) and *Saccharum spontaneum* L. (energy cane—variety VX2—experiment II)). The energy cane variety was obtained from Vignis and classified as type II due to its high fiber content (> 28%) and low sugar content in the form of sucrose (< 6%)^26^. All plant studies were carried out following relevant institutional, national, or international guidelines and regulations. Our research was not conducted with endangered species and was conducted following the Declaration of IUCN Policy on Research Involving Endangered Species.

Experiments were carried out in two stages. The first consisted of the formation of seedlings cultivated in pots filled with inert substrate. During this period, part of the seedlings received Si treatments from 10 to 70 days after sprouting while the other seedlings did not receive Si. The second stage consisted of transplanting these seedlings to pots filled with samples of Entisol (Quartzipsamment). During this stage, part of the seedlings received Si treatments up to 80 days after transplanting. Water regimes covered the period from 7 to 160 days after transplanting, with the subsequent completion of experiments.

### Treatments and experimental design

Treatments were arranged in a 2 × 2 factorial scheme in both experiments. The first factor consisted of the absence (-Si) or presence of Si (+ Si). Si was applied via fertigation and foliar spraying during seedling formation and after transplanting. The second factor consisted of soil water regimes: 70% (no deficit—control) and 30% (severe water deficit—WD) of the soil water retention capacity (WRC), applied only after transplanting the seedlings. Plots were arranged in randomized blocks with 5 replications.

Presprouted seedlings were produced in the first stage of the experiment. To this end budded setts (5 ± 0.5 cm) were planted in seedling production trays filled with fine vermiculite. The nutrient solution used was Hoagland and Arnon^27^ with a change in iron concentration in the Fe–EDDHA source to 368 μmol L^−1^ as indicated by Cavalcante et al.^28^ In order to avoid substrate salinization, the concentration of the nutrient solution was maintained at 25% dilution during the first week of cultivation, increasing to 50% on the second week until the end of the seedling formation phase. A saturation test was carried out to determine the volume of solution to be applied, with a volume of 10 mL per cell being sufficient to saturate the substrate, avoiding loss by leaching. The pH value of the solution was adjusted to 5.5 ± 0.2 with a solution of Hydrochloric acid (HCl) or Sodium hydroxide (NaOH), both at 1 mol L^−1^.

The source of soluble Si used was sodium and potassium silicate stabilized with sorbitol (113.4 g L^−1^ of Si, 18.9 g L^−1^ of K_2_O, 100 mL L^−1^ of sorbitol, and pH 11.8) at 2.5 mmol L^−1^. This Si concentration does not induce polymerization as this phenomenon only starts at a concentration greater than 3 mmol L^−1^^21^. Fifteen Si applications were performed at 4 day intervals, starting 10 days after emergence (DAE) of sprouts (S 1). More specifically, 10 mL of Si per seedling was applied via fertigation in the substrate (to induce root uptake) and 1.5 mL of Si per seedling was applied via foliar spraying (to induce foliar uptake). The pH value of the Si solution was adjusted to 5.5 ± 0.2. The amount of potassium present in the Si source was balanced in the treatments without the element by using a 1 mol L^−1^ potassium chloride solution with root application and foliar spraying. In each condition of Si supply (+ Si and -Si) 50 seedlings of each species were used, being selected for transplanting those with the most uniform length and diameter.

The second phase of the experiment started after Si fertilization, at 70 DAE. On that occasion, 20 dm^3^ pots (surface area = 962 cm^2^) were filled with Entisol (Quartzipsamment) samples^29^ collected from the surface horizon.

(Ap). Soil chemical analysis was carried out for fertility purposes according to the method described by Raij et al.^30^. The results are as follows: pH (CaCl_2_): 4.3, organic matter: 9 g dm^−3^, P (res): 2 mg dm^−3^, B: < 0.12 mg dm^−3^, Cu: 0.2 mg dm^−3^, Fe: 9 mg dm^−3^, Mn: 1.7 mg dm^−3^, Zn: 0.4 g dm^−3^, Ca: 3 mmol_c_ dm^−3^, Mg: 1 mmol_c_ dm^−3^, K: 0.3 mmol_c_ dm^−3^, H + Al: 16 mmol_c_ dm^−3^, Sum of bases (Ca + Mg + K) (SB): 4 mmol_c_ dm^−3^, Cation exchange capacity (CEC): 20.3 mmol_c_ dm^−3^, and Base saturation (V: SBx100/CEC): 21%. Silicon content was 1 mg dm^−3^, being determined by using calcium chloride at 0.01 mol L^−1^ as extractor according to the method of Korndörfer et al.^31^.

Limestone was applied (relative power of total neutralization: 125%, CaO: 48%, MgO: 16%) thirty days before transplanting the seedlings to raise V to 60%. The soil sample was properly mixed and sustained at 70% WRC with to induce the limestone reaction. After this period, fertilizer was applied to the soil using 250 mg dm^−3^ of N and K and 150 mg dm^3^ of P as ammonium sulfate, potassium chloride, and triple superphosphate, respectively. Triple superphosphate was applied in a single dose and incorporated into the soil volume. In turn, N and K were applied via fertigation in five doses of 50 mg dm^−3^ starting four days after transplanting, with seven-day intervals. Moreover, 5 mg dm^−3^ of Zn as zinc sulfate and 2 mg dm^−3^ of B as boric acid were applied via fertigation in a single dose along with the first application of N and K fertilizers.

At the time of transplanting, the seedlings had six fully developed leaves and the cut was made at 30 cm from the sheath of the first newly developed leaf. Therefore, almost one-third of the leaves were removed. This is a common practice in seedling nurseries since it helps decreasing water vapor loss by transpiration when transplanting the seedlings to soil^32^. The seedlings were transplanted to pots filled with soil and maintained at 70% WRC for seven days, being subsequently subjected to soil water regimes.

After transplanting, five more Si fertigations were carried out with soil application and foliar spraying at 20, 35, 50, 65, and 80 days after transplanting (S 1). Silicon application via fertigation was performed by simulating a 5 mm irrigation blade of silicate solution, with 481 mL of solution per pot being applied via roots considering an area of 962 cm^2^. Silicon concentration was 2.5 mmol L^-1^, corresponding to 33.7 mg of Si per pot and equivalent to 3.5 kg ha^−1^ of Si per application. Foliar spraying was carried out using a manual sprayer to ensure leaf coverage without any runoff. The pH value of the fertigated solution was adjusted to 5.5 ± 0.2. The amount of potassium present in the Si source was equilibrated in plants that did not receive Si solution by using a 1 mol L^−1^ potassium chloride solution with application via root and foliar spraying.

Soil water regimes were determined on the basis of microporosity values ​​obtained by the tension table method with a 60 cm water column. To this end, undisturbed soil samples were collected using a volumetric ring with volume (v) of 98.125 cm^3^. Subsequently, the samples were saturated with water for 24 h, being placed on the tension table for 72 h, followed by weight determination (a). After that, the samples were dried in an oven at 110 °C for 24 h and the weight was determined again (b). Total microporosity (Mi): ((a-b)/v) was equivalent to 100% WRC^33^. However, the ideal water condition is 70% of this value, which would allow 70% of the micropores to be filled with available water and the remaining 30% to be filled with air, maintaining root gas exchange^34^. Thus, soil water regimes for sugarcane were: no water deficit (control) (70% WRC) and severe water deficit (WD) (30% WRC) as indicated by Teixeira et al.^35^.

Irrigation management was carried out daily so that soil moisture within each treatment would not alter the biological response of the plant. Thus, the soil mass used to fill each pot was strictly controlled, and pot mass was also defined to be subtracted from the total mass. For that, the mass restoring method was used considering water losses by soil evaporation and plant transpiration, which were controlled daily by weighing the pots. This ensured that the plants were maintained at the WRC levels proposed in the treatments, as adjustments were made in all pots. The same irrigation frequency was used for both species and for the irrigation treatments, that is, all samples were weighed daily.

At the time of water replacement, 70% WRC pots had approximately 60% WRC, and 30% WRC pots had 25% WRC. Water variation was greater in 70% WRC pots because of the higher transpiration losses due to plant growth. In addition, irrigations were always carried out at the same time of day (at 5 p.m.). Preliminary tests showed that water loss was more accentuated between 2:00 and 5:00 p.m.. Therefore, although there was variation, it only occurred during a period of 3 h per day.

Silicon application in treatments with water deficit ended 160 days after transplanting the seedlings to pots filled with soil (initial culm formation). The plants were evaluated as described below.

### Analyses

#### Quantum efficiency of photosystem II (Fv/Fm)

Quantum efficiency of PSII was analyzed between 8 a.m. and 1 p.m., on the first fully developed leaf, using a portable fluorometer (Os30P + , Opti-Sciences Inc., USA)^36^.

#### Chlorophyll and carotenoid content

Five leaf discs of 15 mg were collected in the middle third of the leaf blade of the first fully developed leaf. Readings were performed on a spectrophotometer (DU640, Beckman, USA) at 663 nm for Chlorophyll *a* (Chl *a*), 647 nm for Chlorophyll *b* (Chl *b*), and 470 nm for carotenoids according to the methodology proposed by Lichtenthaler^37^. Contents were determined on the basis of fresh matter.

#### Photosynthetic parameters

Photosynthetic parameters were assessed in the middle third of the first fully developed leaf, avoiding the midrib. Gas exchange parameters were measured with a portable photosynthesis analyzer (LcPro-SD, ADC BioScientificLtd., Hoddesdon, UK). Data were collected in the morning, between 9 and 11 a.m. Gas exchange measurements were taken at a constant light intensity of 1800 µmol m^−2^ s^−1^ emitted by a blue-red LED light source, under natural CO_2_ conditions (403–428 ppm). Leaf temperature was maintained at 30 ± 0.5 °C.

Net photosynthesis rate (A), leaf transpiration (E), stomatal conductance (Gs), and intracellular CO_2_ concentration (Ci) were determined after stabilization (3–5 min). Instantaneous water use efficiency (WUE) was calculated by the ratio between A and E. In turn, instantaneous carboxylation efficiency (ICE) was calculated by the ratio between A and Ci.

#### Electrolyte leakage index

Ten leaf discs (26.4 mm^2^ each) were collected from the first fully developed leaf in two periods (5 a.m. and 2 p.m.). The discs were emerged in deionized water for 2 h and the electrical conductivity (EC_1_) of the solution was read using a conductivity meter (AK51, Akso, Brazil). Samples were autoclaved at 121 °C for 20 min, and final electrical conductivity (EC_2_) was determined after cooling. Electrolyte leakage index was determined from the formula: EC_1_/EC_2_ × 100^38^.

#### Leaf water potential (Ψw)

Leaf water potential (Ψw) was determined in the middle third of the blade of the second fully developed leaf using a Scholander pressure chamber (3000F01, Soil Moisture Equipment, USA). Pressure was applied until exudation from the cut made on the leaf petiole^39^. Measurements took place between 5 a.m. and 1 p.m.

#### Relative water content

Ten discs (26.4 mm^2^) were collected from the first fully developed leaf in two periods (6 a.m. and 2 p.m.). The discs were immediately weighed to determine fresh weight (FW). The samples were then rehydrated in deionized water for 6 h to obtain turgid weight (TW) and dried in a forced air circulation oven (TE-394/3-MP, Tecnal, Brazil) at 80 °C for 24 h to determine dry weight (DW). The values were determined by the equation proposed by Barrs and Weatherley: [(FW-DW)/(TW-DW)] × 100^40^.

#### Phenolic content

Total phenolic content was determined using 0.1 g of fresh matter from the second fully developed leaf. This plant material was immediately weighed and immersed in concentrated methanol, being sustained in the dark for 3 h. After extraction, a colorimetric reaction of total phenols was induced with 2 N Folin-Ciocalteu reagent, allowing reacting for three minutes, and 20% sodium carbonate, allowing reacting for two hours. Subsequently, absorbance was read on a spectrophotometer (B442, Micronal, Brazil) at a wavelength of 765 nm. Phenolic content was determined using a standard curve with gallic acid, being expressed as gallic acid equivalent (GAE) 100 g^−1^^41^.

#### Proline content

Proline content was determined in samples from the third fully developed leaf, which were collected and immediately stored in a freezer at − 80 °C. Ninhydrin reaction was used following the method proposed by Bates et al.^42^. Proline concentration was measured at 520 nm in a spectrophotometer (Beckman DU 640). The values were estimated using a standard curve, being expressed in μmol g^−1^ FW.

#### Hydrogen peroxide (H_2_O_2_)

Hydrogen peroxide (H_2_O_2_) content was determined in samples from the third fully developed leaf, which were collected and immediately stored in a freezer at − 80 °C. Reaction with potassium iodide followed the method of Alexieva et al.^43^. The material was read on a spectrophotometer (DU640, Beckman, USA) at 390 nm, and H_2_O_2_ content was determined using a standard curve. The results were expressed in μMol g^−1^ FW.

#### Lipid peroxidation

Lipid peroxidation was determined in samples from the third fully developed leaf, which were collected and immediately stored in a freezer at − 80 °C. The determination of the content of substances reactive to thiobarbituric acid followed Heath and Packer^44^ . Malondialdehyde (MDA) content was determined on a spectrophotometer (DU640, Beckman, USA) at 535 and 600 nm. Data were calculated using an extinction coefficient of 1.55 × 10^−5^ mol^−1^ cm^−1^^45^ . The results were expressed in nMol g^−1^ FW.

#### Antioxidant enzymes

For enzymatic analysis, protein extracts were initially obtained^46^ using samples from the third fully developed leaf that were collected and immediately stored in a freezer at − 80 °C, following Gomes-Junior et al.^46^. The samples were macerated in liquid N_2_ and homogenized in potassium phosphate buffer containing ethylene diaminetetraacetic acid, dithiothreitol, and polyvinylpolypyrrolidone. The homogenate was centrifuged and the supernatant was stored in aliquots at − 80 °C. Total protein content was determined according to the method of Bradford^47^.

##### Catalase (CAT, EC 1.11.1.6)

Catalase activity was determined from protein extracts using the method described by Kraus et al.^48^ modified by Azevedo et al.^49^. The activity was determined by monitoring the degradation of H_2_O_2_ at 240 nm for 1 min. The results were expressed in μmol min^−1^ mg^−1^ protein.

##### Superoxide dismutase (SOD, EC 1.15.1.1)

Superoxide dismutase activity was determined according to Giannopolitis and Ries^50^ based on the measurement of inhibition of the photochemical reduction of blue nitro chloride tetrazolium. The procedure was carried out in a reaction chamber illuminated by a 15 W fluorescent lamp, at 25 °C. Measurement was performed using a spectrophotometer at 560 nm, and the results were expressed in SOD U mg^−1^ protein.

##### Ascorbate peroxidase (APX, EC 1.11.1.11)

Ascorbate peroxidase activity was determined by monitoring the oxidation rate of ascorbate at 290 nm following the method of Moldes et al.^51^. The results were expressed in nMol of ascorbate min^−1^ mg^−1^ protein.

#### Number of tillers, number of green leaves, percentage of dry leaves, and leaf area

Number of tillers and number of green leaves were determined by counting. The percentage of dry leaves was obtained from the ratio of the number of dry leaves by the total number (dry + green) of leaves, multiplied by 100. Leaf area was measured using a leaf area meter (L-3100, LiCor, USA).

#### Dry mass production

Plants were separated into leaves and culms and washed in running water with detergent solution (0.1% v/v), HCl solution (0.3% v/v), and deionized water. The plant material was dried in a forced air circulation oven (TE-394/3-MP, Tecnal, Brazil) (65 ± 5 °C) to constant mass in order to obtain the dry mass of each part of the plant.

#### Si analyses

Si content in the leaves and culms were determined by extracting this element according to the methodology of Kraska and Breitenbeck^52^. Reading was performed using a spectrophotometer (B442, Micronal, Brazil) at 410 nm, as indicated by Korndörfer et al.^31^. Silicon accumulation in the leaves and culms of plants was calculated on the basis of Si content and dry mass.

### Statistical analysis

The experiments were analyzed independently for both species. In order to normalize variance, the percentage of electrolyte leakage index, percentage of dry leaves, and relative water content were transformed to the arc sine = $\surd \frac{x}{100}$. The data underwent bidirectional analysis of variance by the F-test (*p* < 0.05) after meeting the assumptions of normality (Shapiro-Wilks W test) and homogeneity of variances (Bartlett test).

Factorial analysis was used for testing the main effects of silicon (Si) supply and soil water regime (WD) and their interactions (Si × WD), where N = 20 experimental units in each experiment (Supplementary file 2). Means were compared by the Tukey test at 5% probability using SAS statistical software (Cary, NC, USA).

### Statement of handling of plants

The authors confirm that the handling of the plants is accordance with the Declaration of IUCN Policy on Research Involving Endangered Species and the Convention on Trade in Endangered Species of Wild Fauna and Flora.

## Results

**Figure 1:**
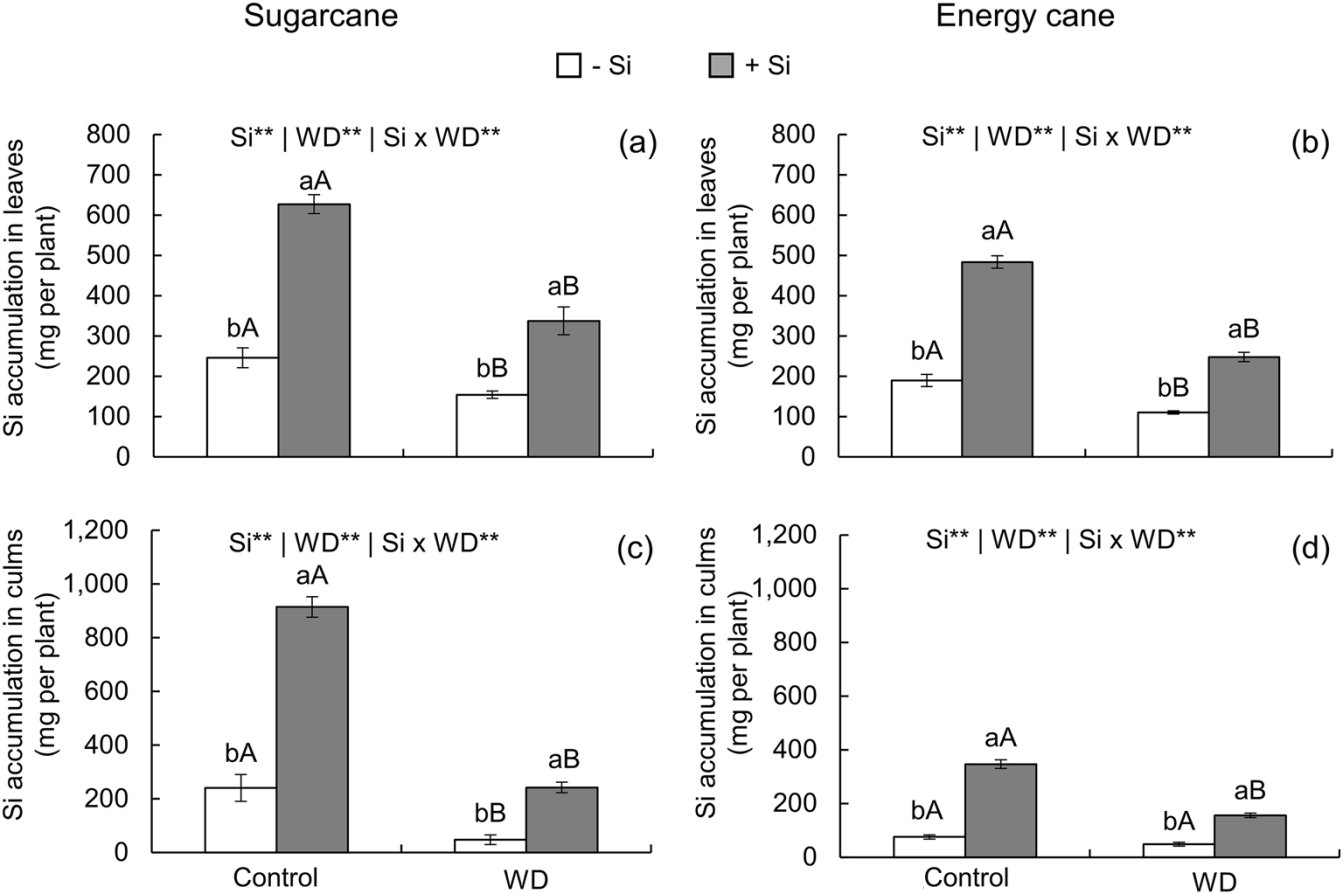
Silicon (Si) accumulation in leaves and culms of sugarcane (**a**, **c**) and energy cane (**b**, **d**) in the absence of Si (-Si) and with Si applied via fertigation associated with foliar spraying (+ Si) under adequate water regime (control) and severe water deficit (WD) for a period of 7 to 160 days after transplanting. ^**^: significant at 1% probability by the F-test. Different lowercase letters indicate differences in relation to Si in the same water regime; different capital letters indicate differences in relation to the water regime in the same conditions of Si supply, both by the Tukey test. Bars represent the standard error of the mean; *n* = 5.

In sugarcane, silicon (Si) application in the seedling formation phase with complementation after transplanting increased the accumulation of this element in leaves and culms by 155 and 280% in the controlled water regime, and by 119 and 400% in the water deficit regime, respectively (Fig. 1a,c). In energy cane, silicon also increased the accumulation of this element in leaves and culms by 155 and 358% in the controlled water regime, and by 125 and 225% under water deficit, respectively (Fig. 1b,d).

In sugarcane, Si accumulated more in the culms than in the leaves of plants that received Si fertigation in association with foliar spraying (Fig. 1a,c). On the other hand, in energy cane plants that received Si application, this element accumulated more in the leaves than in the culms (Fig. 1b,d). Severe water deficit decreased Si accumulation in the leaves and culms of sugarcane under both Si supply conditions (Fig. 1a,c). In energy cane, water deficit decreased Si accumulation in the leaves and culms of plants under + Si, and only in the leaves of plants under -Si (Fig. 1b,d).

**Figure 2:**
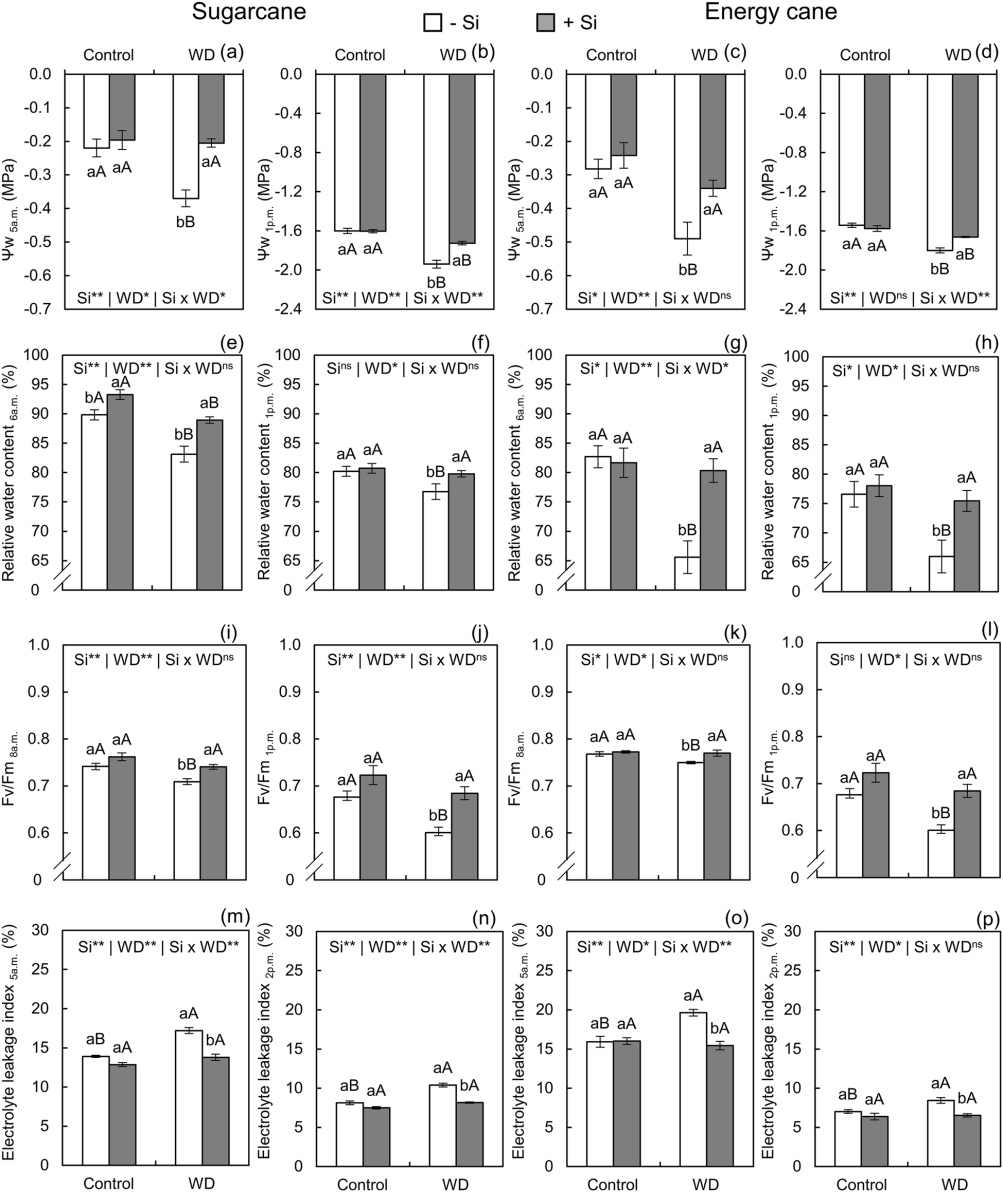
Leaf water potential (Ψw), relative water content, quantum efficiency of photosystem II (Fv/Fm), and electrolyte leakage index in sugarcane (**a**, **b**, **e**, **f**, **i**, **j**, **m**, **n**) and energy cane (**c**, **d**, **g**, **h**, **k**, **l**, **o**, **p**) in the absence of Si (-Si) and with Si applied via fertigation associated with foliar spraying (+ Si) under adequate water regime (control) and severe water deficit (WD) for a period of 7 to 160 days after transplanting. ^**^ and ^*^: significant at 1 and 5% probability, respectively; and ^ns^: not significant by the F-test. Different lowercase letters indicate differences in relation to Si in the same water regime; different capital letters indicate differences in relation to the water regime in the same conditions of Si supply, both by the Tukey test. Bars represent the standard error of the mean; *n* = 5.

In sugarcane (Fig. 2a) and energy cane (Fig. 2c) plants grown under -Si, Ψw assessed at dawn (at 5 a.m.) decreased under water deficit in relation to the controlled water regime. However, Si supply increased Ψw in the water deficit condition in relation to -Si, even remaining similar to that of plants of both species grown under adequate water conditions (Fig. 2a,c). At the hottest period of the day (at 1 p.m.), the imposition of water deficit decreased Ψw in relation to the controlled water regime, in both + Si and -Si, for both species (Fig. 2b,d).

In -Si, water deficit decreased relative leaf water content in the assessments at 6 a.m. and 2 p.m. in sugarcane and energy plants in relation to adequate water regime. Silicon enrichment in plants of both species reduced leaf water content loss in the assessments at 6 a.m. and 2 p.m. in the severe water deficit regime. The values were even similar to those of unstressed plants, except for the assessment at 6 a.m. in sugarcane (Fig. 2e-h).

Quantum efficiency of PSII, expressed by Fv/Fm values assessed at 8 a.m. and 1 p.m., was lower in plants of both species that did not receive Si supply in the condition of severe water deficit in relation to the controlled water condition (Fig. 2i-l). Silicon application in relation to its absence increased the efficiency of PSII in sugarcane and energy cane plants under water deficit. The values were similar to that of plants under controlled water regime in the two evaluation periods (Fig. 2i-l).

In -Si, water deficit in relation to the controlled water regime increased cellular electrolyte leakage index in the assessments at 5 a.m. and 2 p.m. for both species. However, Si supply in relation to its absence prevented an increase in electrolyte leakage index in sugarcane and energy cane plants under severe water deficit in the two evaluation periods (Fig. 2m-p). This result is similar to that of sugarcane (Fig. 2m,n) and energy cane (Fig. 2o, p) plants treated without water deficit.

**Figure 3:**
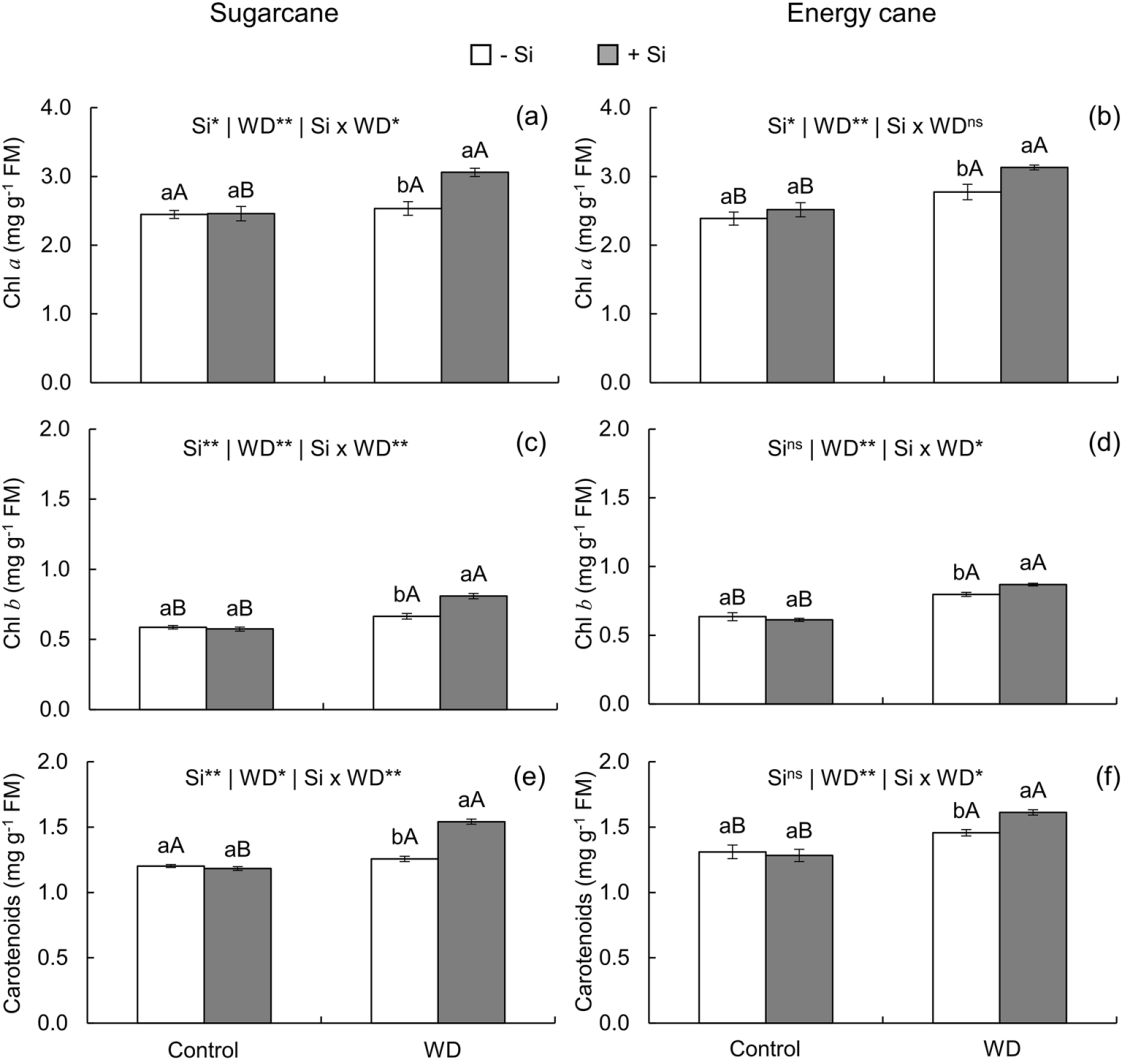
Chlorophyll *a* (Chl *a*), chlorophyll *b* (Chl *b*), and carotenoid content in sugarcane (**a**, **c**, **e**) and energy cane (**b**, **d**, **f**) in the absence of Si (-Si) and with Si applied via fertigation associated with foliar spraying (+ Si) under adequate water regime (control) and severe water deficit (WD) for a period of 7 to 160 days after transplanting. ^**^ and ^*^: significant at 1 and 5% probability, respectively; and ^ns^: not significant by the F-test. Different lowercase letters indicate differences in relation to Si in the same water regime; different capital letters indicate differences in relation to the water regime in the same conditions of Si supply, both by the Tukey test. Bars represent the standard error of the mean; *n* = 5. FW = fresh weight.

**Figure 4:**
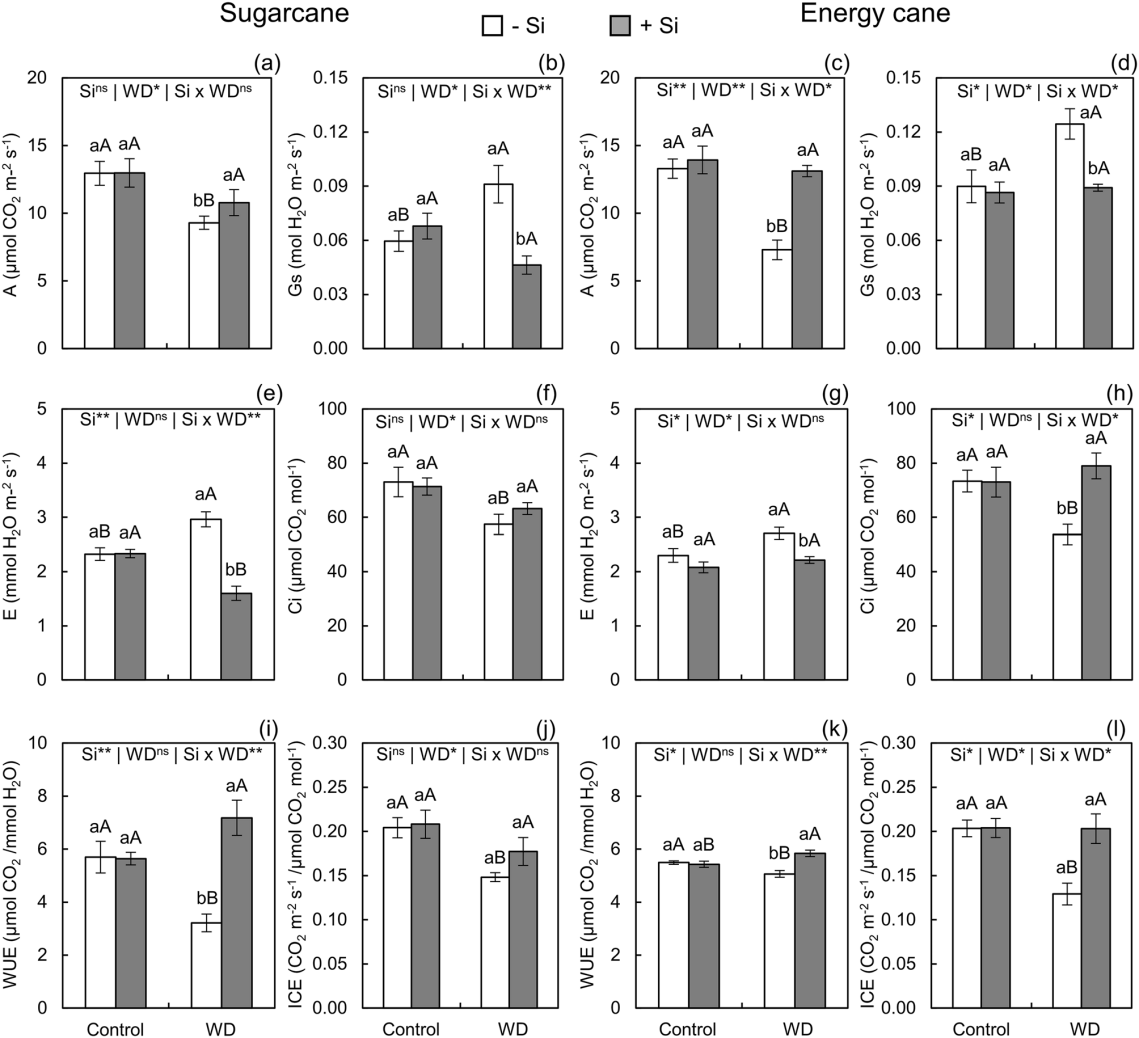
Photosynthesis rate (A), stomatal conductance (Gs), intracellular CO_2_ concentration (Ci), leaf transpiration (E), water use efficiency (WUE), and instantaneous carboxylation efficiency (ICE) in sugarcane ( **a**, **b**, **e**, **f**, **i**, **j**) and energy cane (**c**, **d**, **g**, **h**, **k**, **l**) in the absence of Si (-Si) and with Si applied via fertigation associated with foliar spraying (+ Si) under adequate water regime (control) and severe water deficit (WD) for a period of 7 to 160 days after transplanting. ^**^ and ^*^: significant at 1 and 5% probability, respectively; and ^ns^: not significant by the F-test. Different lowercase letters indicate differences in relation to Si in the same water regime; different capital letters indicate differences in relation to the water regime in the same conditions of Si supply, both by the Tukey test. Bars represent the standard error of the mean; *n* = 5.

In sugarcane plants grown under water deficit and -Si, Chl *a* content remained unchanged while Chl *b* content increased in relation to the values of plants grown under controlled water condition and -Si (Fig. 3a,b). Moreover, Chl *a* and Chl *b* contents increased in energy cane plants grown without Si application under severe water deficit in relation to adequate water regime (Fig. 3c,d). However, silicon supply increased Chl *a* and Chl *b* contents in plants of both species under severe water deficit (Fig. 4a-d).

Comparing with the controlled water regime, the imposition of water deficit to the soil of plants that did not receive Si increased carotenoid content in energy cane, although this variable did not change in sugarcane under these conditions. However, the application of the beneficial element increased the content of this pigment in both species under water restriction in relation to plants grown under controlled water regime (Fig. 3e,f).

Water deficit decreased A in relation to the controlled water regime in sugarcane and energy cane plants that did not receive Si. However, under water deficit, Si supply reduced A losses in the plants, with values even similar to those of plants of both species under controlled water conditions (Fig. 4a,c).

It is noteworthy that Gs increased in plants of both species grown without Si fertilization under water deficit in relation to water sufficiency. Notwithstanding, Si supply decreased the Gs of sugarcane and energy cane plants under severe water deficit, reaching values similar to those of plants without water stress (Fig. 4b,d).

Regarding -Si, plants grown under water deficit had higher E than plants grown under controlled water conditions. However, Si enrichment in plants decreased the E of both species under water deficit in relation to the absence of Si application. This Si-induced decrease in E reduced transpiration rates in sugarcane while energy cane plants had values similar to those of unstressed plants (Fig. 4e,g).

In the absence of Si, Ci decreased in sugarcane and energy cane plants under water deficit in relation to those under controlled water regime. However, Si supply increased the Ci of sugarcane plants under water deficit. This increase was enough for their Ci concentration to equal those of unstressed plants (Fig. 4f,h).

The water use efficiency (WUE) of plants grown without Si application decreased in sugarcane and energy cane plants under water deficit in relation to plants grown under adequate water regime. However, WUE benefited from Si supply in plants under water deficit, increasing by 123 and 15.2% in sugarcane and energy cane, respectively, in relation to plants under -Si (Fig. 4i,k).

Plants of both species grown without Si supply had lower instantaneous carboxylation efficiency (ICE) in the water deficit regime than in the controlled water regime. However, nutrition with Si increased ICE in plants under water deficit, reaching values similar to those of plants of both species without water stress (Fig. 4j,l).

**Figure 5:**
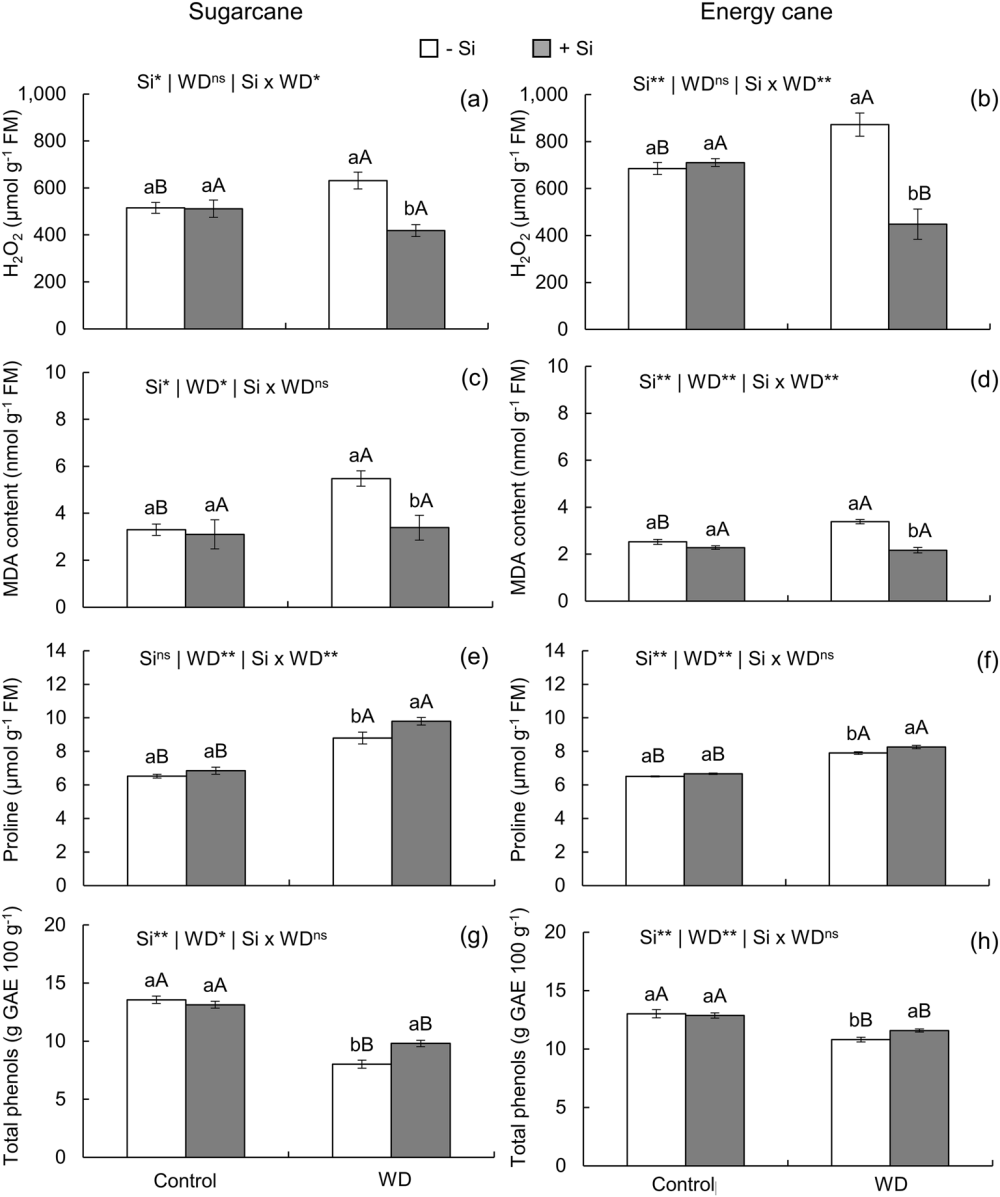
Hydrogen peroxide (H_2_O_2_), malondialdehyde (MDA), proline, and total phenolic content in sugarcane (**a**, **c**, **e**, **g**) and energy cane (**b**, **d**, **f**, **h**) in the absence of Si (-Si) and with Si applied via fertigation associated with foliar spraying (+ Si) under adequate water regime (control) and severe water deficit (WD) for a period of 7 to 160 days after transplanting. ^**^ and ^*^: significant at 1 and 5% probability, respectively; and ^ns^: not significant by the F-test. Different lowercase letters indicate differences in relation to Si in the same water regime; different capital letters indicate differences in relation to the water regime in the same conditions of Si supply, both by the Tukey test. Bars represent the standard error of the mean; *n* = 5. FW = fresh weight. GAE = gallic acid equivalent.

Absence of Si supply increased H_2_O_2_ content (Fig. 5a,b) and lipid peroxidation—expressed by MDA content (Fig. 5c,d)—in plants of both species grown under severe water deficit in relation to the controlled water regime. However, Si supply decreased H_2_O_2_ content and lipid peroxidation in plants of both species under water deficit (Fig. 5a-d). The Si-induced decrease in H_2_O_2_ and MDA made these concentrations similar to those of unstressed plants in sugarcane (Fig. 5a, c, d), but with lower H_2_O_2_ in energy cane (Fig. 5b).

Proline content increased with water deficit under both Si supply conditions in sugarcane and energy cane plants. However, Si supply accentuated the increase in proline content in plants under water deficit, reaching values higher than those of plants of both species grown under -Si (Fig. 5e, f).

Cultivation of sugarcane and energy cane plants under severe water deficit decreased total phenolic content regardless of Si nutrition in relation to cultivation under controlled water regime. However, Si supply decreased the losses in total phenolic content in plants of both species under water deficit (Fig. 5g,h).

**Figure 6:**
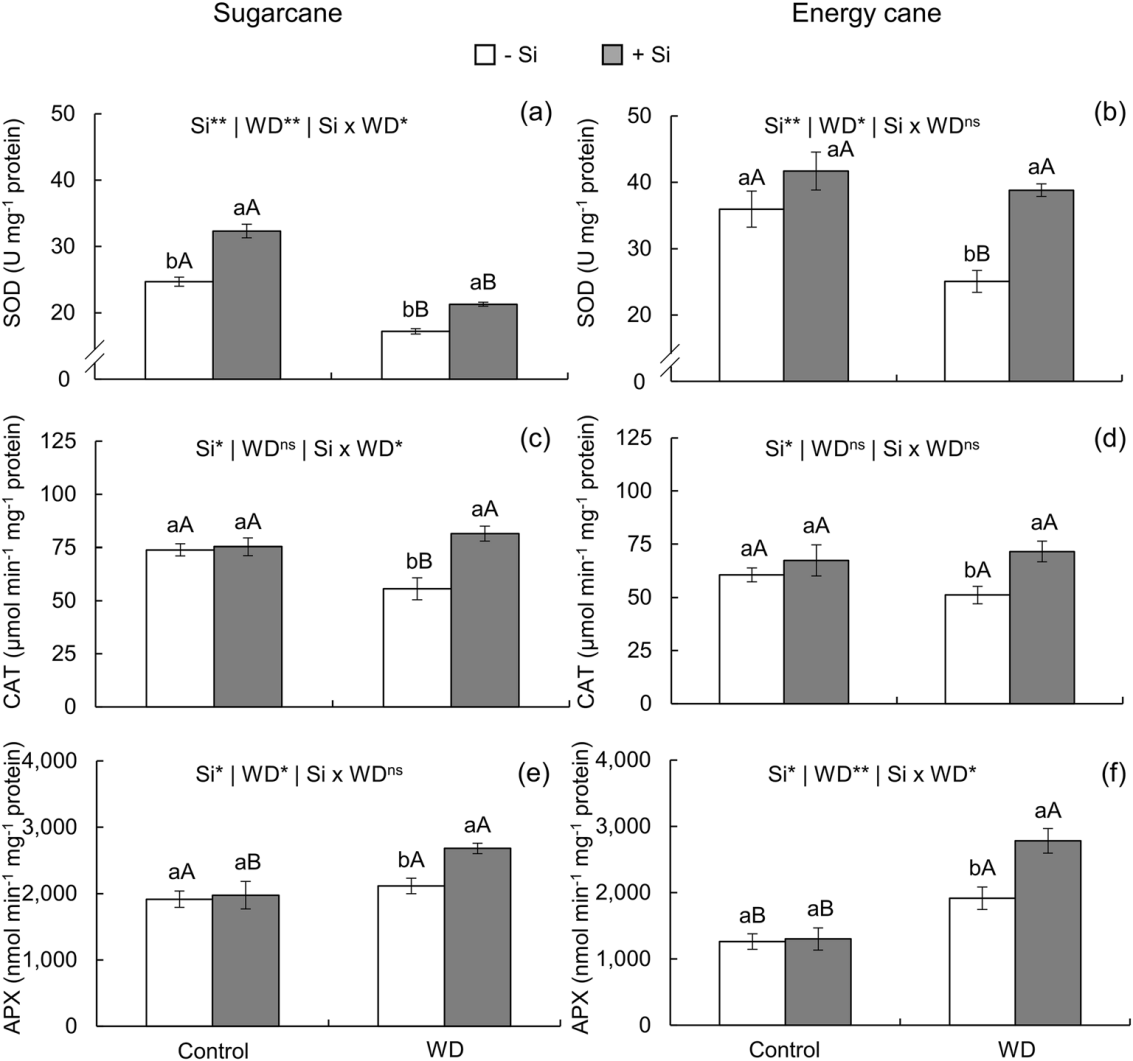
Specific activity of superoxide dismutase (SOD), catalase (CAT), and ascorbate peroxidase (APX) in sugarcane (**a**, **c**, **e**) and energy cane (**b**, **d**, **f**) in the absence of Si (-Si) and with Si applied via fertigation associated with foliar spraying (+ Si) under adequate water regime (control) and severe water deficit (WD) for a period of 7 to 160 days after transplanting. ^**^ and ^*^: significant at 1 and 5% probability, respectively; and ^ns^: not significant by the F-test. Different lowercase letters indicate differences in relation to Si in the same water regime; different capital letters indicate differences in relation to the water regime in the same conditions of Si supply, both by the Tukey test. Bars represent the standard error of the mean; *n* = 5.

Superoxide dismutase (SOD) activity decreased with the imposition of severe water deficit in sugarcane and energy cane (Fig. 6a-b) plants that did not receive Si fertilization. Silicon supply totally reversed this decrease in energy cane plants (Fig. 6b) and partially reversed it in sugarcane (Fig. 6a), as + Si plants had higher SOD activity under water deficit than -Si plants. However, SOD activity increased even in unstressed sugarcane plants that received Si in relation to -Si plants in the same water regime (Fig. 6a).

Cultivation of sugarcane and energy cane plants under severe water deficit decreased catalase (CAT) activity in relation to the controlled water regime. However, Si supply via fertigation in association with foliar spraying increased CAT activity in plants of both species, reaching values similar to those of unstressed plants (Fig. 6c-d).

In relation to the controlled water regime, ascorbate peroxidase (APX) activity increased in plants under severe water deficit and without Si supply only in energy cane, remaining unchanged in sugarcane. Silicon fertilization intensified the increase in APX activity in plants of both species under water deficit in relation to unstressed plants (Fig. 6e-f).

**Figure 7:**
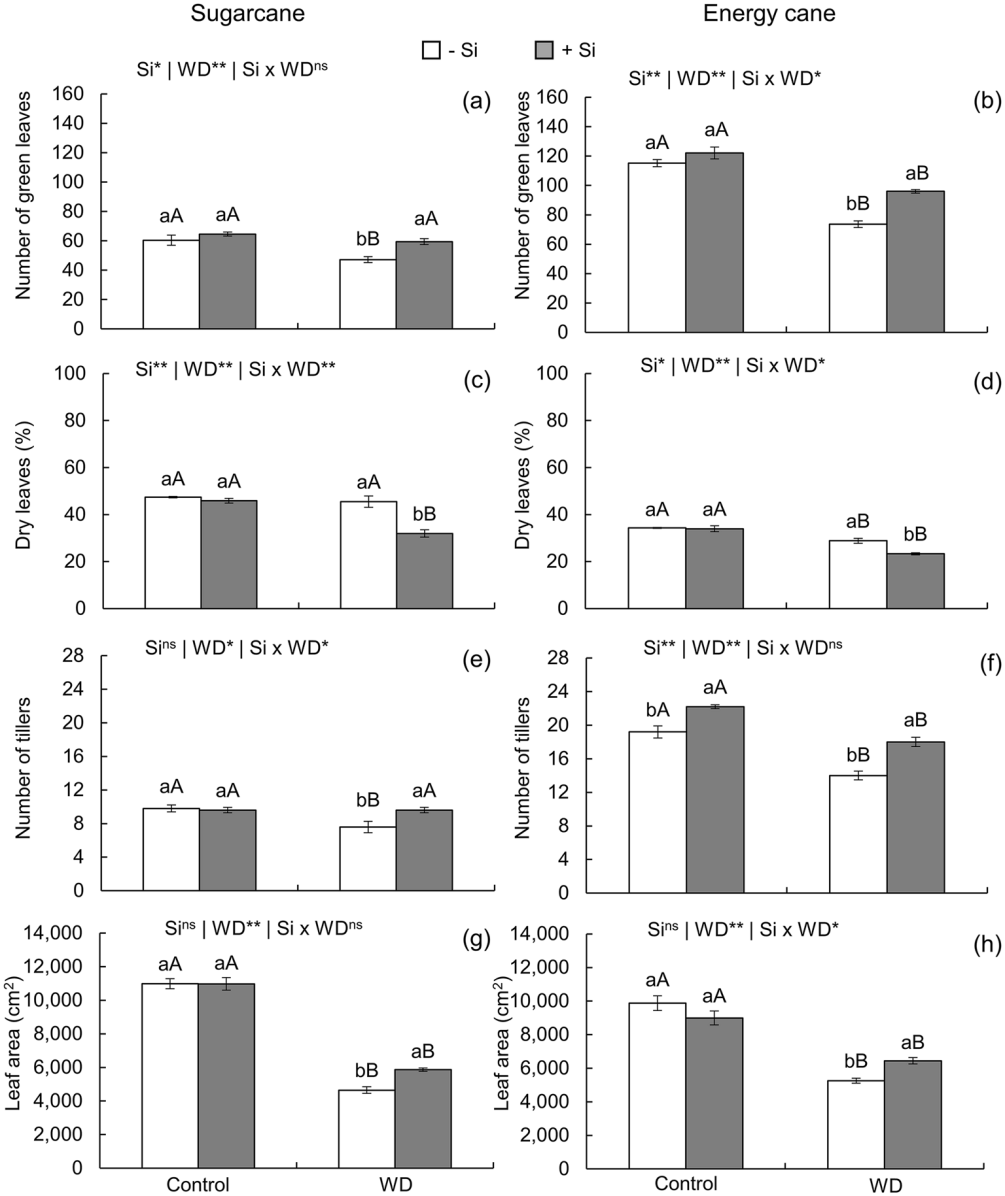
Number of green leaves, percentage of dry leaves, number of tillers, and leaf area in sugarcane (**a**, **c**, **e**, **g**) and energy cane (**b**, **d**, **f**, **h**) in the absence of Si (-Si) and with Si applied via fertigation associated with foliar spraying (+ Si) under water adequate regime (control) and severe water deficit (WD) for a period of 7 to 160 days after transplanting. ^**^ and ^*^: significant at 1 and 5% probability, respectively; and ^ns^: not significant by the F-test. Different lowercase letters indicate differences in relation to Si in the same water regime; different capital letters indicate differences in relation to the water regime in the same conditions of Si supply, both by the Tukey test. Bars represent the standard error of the mean; *n* = 5.

In the absence of Si, severe water deficit reduced the number of green leaves of both species. However, nutrition with Si provided a beneficial effect that increased the number of green leaves of plants under water deficit (Fig. 7a, b), with sugarcane showing values similar to those of plants grown under controlled water regime (Fig. 7a).

The percentage of dry leaves decreased in sugarcane plants under water deficit and without Si supply, remaining unchanged in energy cane plants under the same conditions in relation to the controlled water regime. However, nutrition with Si decreased the percentage of dry leaves in plants of both species under water deficit in relation to the -Si condition (Fig. 7c, d).

In the absence of Si supply, the number of tillers decreased for both species under severe water deficit regime in relation to water sufficiency. However, under water deficit, fertigation with Si increased the number of sugarcane and energy cane tillers (Fig. 7e, f), with sugarcane showing values even similar to those of plants under adequate moisture (Fig. 7e). In energy cane plants, the beneficial effect of Si in increasing tillering also occurred in plants under controlled water regime (Fig. 7f).

Leaf area decreased in plants of both species under severe water deficit, regardless of Si nutrition, in relation to plants under an adequate water regime (Fig. 7g, h). However, Si application to seedlings with complementation after transplanting increased leaf area in sugarcane and energy cane plants grown under water deficit (Fig. 7g, h).

**Figure 8:**
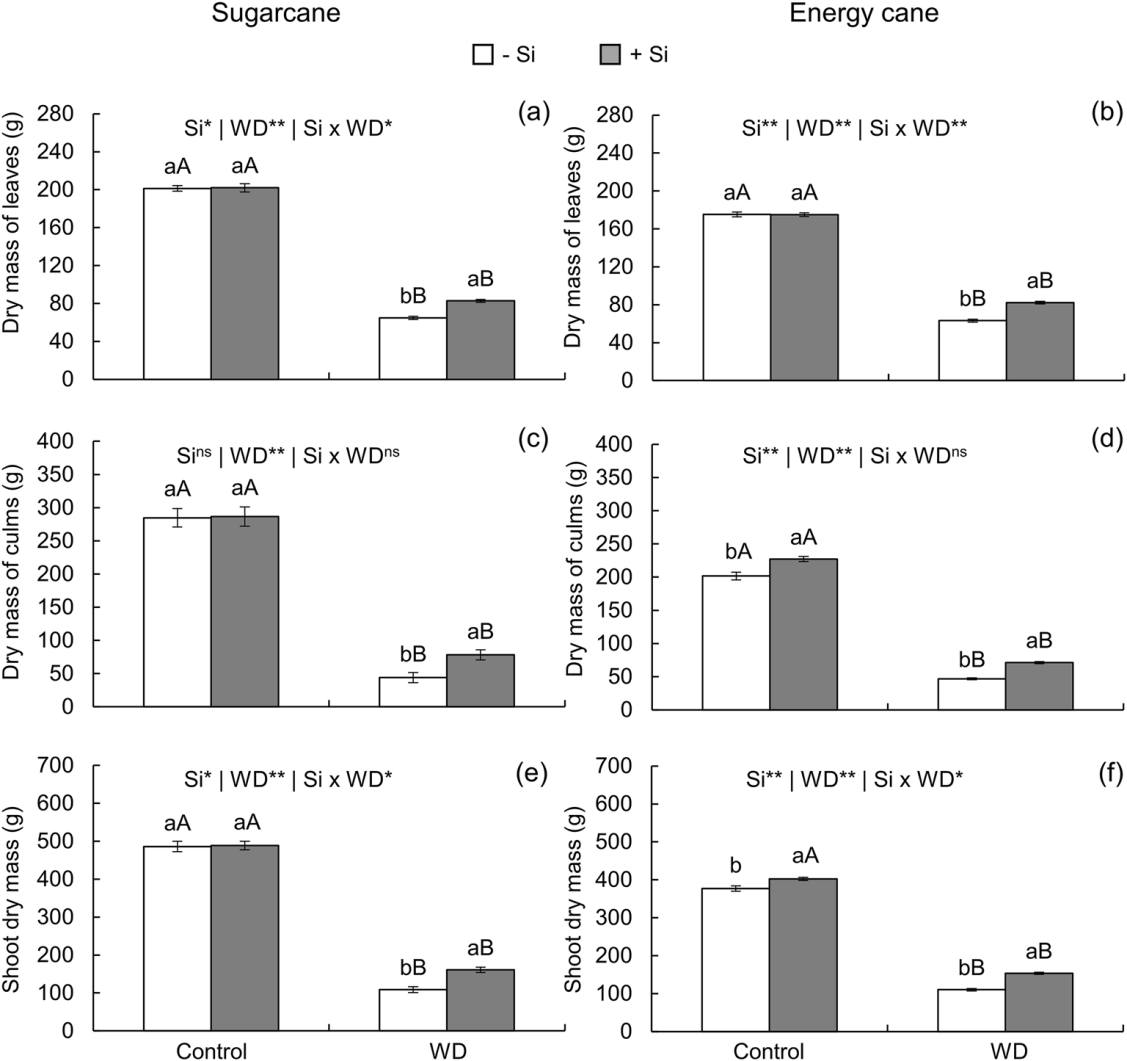
Leaf dry mass, culm dry mass , and shoot dry mass of sugarcane (**a**, **c**, **e**) and energy cane (**b**, **d**, **f**) in the absence of Si (-Si) and with Si applied via fertigation associated with foliar spraying (+ Si) under adequate water regime (control) and severe water deficit (WD) for a period of 7 to 160 days after transplanting. ^**^ and ^*^: significant at 1 and 5% probability, respectively; and ^ns^: not significant by the F-test. Different lowercase letters indicate differences in relation to Si in the same water regime; different capital letters indicate differences in relation to the water regime in the same conditions of Si supply, both by the Tukey test. Bars represent the standard error of the mean; *n* = 5.

In the cultivation of plants under severe water deficit, the dry mass of leaves (Fig. 8a, b), culms (Fig. 8c, d), and shoots (Fig. 8e, f) decreased until initial culm formation in both species. However, Si supply partially reduced these losses, increasing the dry mass of leaves, culms, and shoots by 28, 78, and 48% in sugarcane, and by 30, 52, and 45% in energy cane, respectively (Fig. 8a-f).

## Discussion

Water deficit is one of the main environmental stresses limiting plant growth and consequently reducing agricultural production^1^. This study evidenced the harmful effect of water deficit by showing that cultivation under a water regime of 30% WRC for a period of 7 to 160 days after transplanting impaired biochemical and physiological variables, decreasing plant growth in both sugarcane and energy cane. The sensitivity of energy cane to water deficit had not yet been reported despite the current use of this crop in the sugar-alcohol sector to expand electricity cogeneration. This use stems from the higher energy conversion of this crop in relation to sugarcane due to its greater dry matter production and high fiber content^53^. These characteristics can provide greater tolerance to environmental stress^26^.

However, the results of this study evidence that severe water deficit, which has been a common scenario in regions with an unstable water regime^54^, affects energy cane growth even at a more advanced stage of development, i.e., initial culm formation.

The cultivation of plants of both species under severe water deficit until initial culm formation impaired growth because this condition induced stress, initially caused by the decrease in leaf water content. Lower water content stemmed from the decrease in Ѱw_5am_ and Ѱw_1pm_ in sugarcane and energy cane plants (Fig. 3a-d). Smaller Ѱw limits leaf water uptake because it alters the tension of xylem solution, which is transmitted through continuous water columns to the roots^13,24^. Concomitantly, Gs (Fig. 4b, d) increased resulting in higher E (Fig. 4e, g) and inducing high water vapor losses. Added to the limitation in water absorption by the roots, this has considerably decreased leaf water content (Fig. 2e-h), affecting water status.

Despite the increase in Gs and E, plants under severe water deficit that did not receive Si application had low internal CO_2_ concentration (Fig. 4f, h) and low instantaneous carboxylation efficiency (ICE) (Fig. 4j, l), which decreased A (Fig. 4a, c) in both plant species. These results suggest that the water deficit damage in inhibiting photosynthesis in the plants under study during initial culm formation is predominantly due to nonstomatal limitations. Some authors reported this effect in plants under severe stress^55^ due to the decrease in total soluble protein and photosynthetic pigments. This has led to photochemical inefficiency^55^ while decreasing the activity of the enzymes phosphoenolpyruvate carboxylase (PEPcase) and ribulose-1,5-bisphosphate carboxylase (Rubisco)^56^. However, although the present study has proven photochemical inefficiency (Fig. 3i-l), the content of photosynthetic pigments such as chlorophylls (Fig. 3a-d) and carotenoids (Fig. 3e, f) was not affected. This suggests limitations in the action of enzymes linked to photosynthetic metabolism caused by increased oxidative stress.

Photosynthetic inhibition increased ROS production due to excess light energy in the chloroplasts, which was induced by the decrease in CO_2_ assimilation rate^57^. The increase in H_2_O_2_ content (Fig. 5a, b) evidenced ROS accumulation in plants under severe water deficit in -Si, which destabilized cell membranes leading to lipid peroxidation, as verified by the higher MDA content (Fig. 5c, d)^58^. This condition was also evidenced by the increase in electrolyte leakage index (Fig. 2m-p), which is a physiological parameter that estimates cell membrane integrity^59^. The imbalance in cellular homeostasis that increased ROS accumulation also compromised the integrity of enzymes, damaging the secondary antioxidant defense system of plants.

The antioxidant defense system of plants comprises enzymatic and nonenzymatic antioxidant systems^60^.Superoxide dismutase acts in the first line of enzymatic elimination of ROS, catalyzing the conversion of O_2_^-^ to H_2_O_2_ within the cell^24^. In this study, SOD activity decreased in plants of both species under severe water deficit and without Si supply (Fig. 6a, b). Antioxidant enzymatic defense also includes H_2_O_2_ detoxification. This process occurs through the action of APX and CAT that regulate cellular redox levels by breaking down H_2_O_2_ molecules^60^. Thus, the activity of these enzymes has been analyzed to assess the tolerance of plants grown under drought conditions^24^.

Severe water deficit decreased CAT activity only in sugarcane (Fig. 6c, d), and increased APX activity only in energy cane (Fig. 6e, f). These results demonstrate that sugarcane has a less efficient enzymatic antioxidant detoxification mechanism than energy cane, that is, the first is more sensitive to water deficit. Ascorbate peroxidase activity was also higher in sorghum plants under water deficit^14^.

In addition to the effects on the action of the enzymatic defense system, severe water deficit affected the content of phenols and proline that act on the nonenzymatic antioxidant defense system. Proline content increased with the imposition of water deficit in both species (Fig. 5g, h), confirming the stress condition. Previous studies report the accumulation of free amino acids in plants under drought^14,17,61^ as a strategy to raise osmotic pressure, thus being an osmoregulatory mechanism^62^.

Water deficit also decreased phenolic content (Fig. 5e, f). Phenols are bioactive substances that act as nonenzymatic antioxidants, interfering with metabolic homeostasis from the direct elimination of active molecular oxygen and H_2_O_2_^63^. Thus, phenols protect cell tissues from the action of ROS, inhibiting lipid peroxidation and maintaining cell integrity^64^. In the present study, phenolic contents were comparatively lower in plants under water deficit than in plants under an adequate water regime. This decrease helps to justify the high accumulation of H_2_O_2_ and MDA in both species under study (Fig. 5a-d). The literature mentions similar results in maize plants^61^.

Biological changes caused by oxidative stress in the plant decreased WUE (Fig. 4i, k), indicating losses in the use of available water. This condition affected the development of plants grown under water deficit until initial culm formation, which can be seen from the decrease in leaf area (Fig. 7g, h). This decrease correlates with increased senescence and leaf abscission, evidenced by the decrease in the number of green leaves (Fig. 7a, b) and by the high percentage of dry leaves (Fig. 7c, d). High leaf senescence results from the curling of leaves and the decrease in cell elongation^65^, which intensified from the high rate of lipid peroxidation. This led to sharp decreases in tillering (Fig. 7e, f) and dry mass production for both species (Fig. 8a-f). Other authors reported the harmful effects of water deficit in presprouted sugarcane seedlings, but for a short period of 3 days^17^ and 30 days after transplanting^66^, Moreover, these effects were restricted to physiological and growth variables.

Therefore, although biological mechanisms vary between species, the sensitivity of sugarcane and energy cane to severe water deficit up to the initial stage of culm formation is noteworthy. This condition demonstrates the need to increase the tolerance of these species given the increase in planting areas with recurrent water deficit and the occurrence of cyclical water shortages in different cropping regions. In this context, the present study proposed to enrich these species with Si in the seedling formation phase with complementation via fertigation in association with foliar spraying after transplanting as a strategy to increase plant resistance under water deficit.

Silicon supply by fertigation in association with foliar spraying provided high uptake of this element in both species (Fig. 1a-d). This showed the feasibility of using Si in the seedling formation phase with complementation after transplanting. The efficiency of Si supply is due to proper handling of the solution by using a concentration that favors the permanence of the element in the monomeric form that is absorbed by plants^11^, that is, below 3 mmol L^−1^^21^. Using an adequate source of Si also improved its efficiency. This study used soluble Si along with sorbitol, which favors the stability of the monomeric forms of the element. Silicon forms organic complexes when binding with this polyol^67^, which contributes to its uptake via roots. Sorbitol also increases the wetting properties of the solution by decreasing the deliquescence point of the leaf surface^68^, delaying drying and contributing to Si uptake. Thus, Si supply via fertigation in association with foliar spraying was efficient in inducing the absorption of this element by leaves and roots.

The proven efficiency of Si fertigation and foliar spraying reinforces its applicability in the seedling formation phase. This technique is already used in nurseries for fertilizer application^32^, allowing low concentrations of the beneficial element to be frequently used. Furthermore, complementation after transplanting is also a possibility considering the expansion of irrigated sugarcane cultivation and the use of rescue irrigation, which occurs after transplanting seedlings^5^. Thus, the soil solution can be enriched with Si from its supply via fertigation, increasing H_4_SiO_4_ concentration in the root zone. Fertigation in association with foliar spraying increases Si accumulation in plants, improving the residual effect of this element. The combination attenuates severe water deficit damage for longer periods of time extending to initial culm formation.

The hypothesis can be accepted that the use of fertigation in association with foliar spraying during seedling formation with complementation after sugarcane and energy cane transplanting allows high Si uptake with longer-lasting residual beneficial effects in the plant that extend up to 160 days of cultivation. This finding has important practical implications as it should expand the use of Si in irrigated sugarcane crops. This is because the technique uses a low amount of Si (close to 20 kg ha^−1^) in relation to techniques using ther sources of lower solubility such as calcium silicate, which requires doses above 1000 kg ha^−1^^20^. Previous reports have already shown the efficiency of Si fertigation in optimizing Si uptake in forage plants^22^ and presprouted sugarcane seedlings^17^.

Increased Si accumulation in plants induced an important biological response for the survival of plants under severe water deficit, improving cell water status. The applications maintained the water potential similar to that of plants without water deficit in the first hours of the day, with variation only at times of higher temperature (Fig. 2a, c). This adjustment in leaf water potential facilitated plant water uptake and increased relative leaf water content (Fig. 2e-h). Higher leaf water content correlates mainly with physical changes caused by the deposition of silica that binds to cellulose in the leaf epidermis and below the cuticle.^11^ This deposition acts as a barrier to water loss, decreasing transpiration. As evidenced in the present study, this occurrence is common in Si-accumulating plants^69^ (Fig. 4e, g). This is because the absorbed monosilicic acid reaches the leaves through the transpiration gradient^11^ and concentrates inducing polymerization as amorphous or biogenic silica being bound to cell wall cellulose, constituting 90% of the absorbed Si^12^.

Plants treated with Si also have higher water content due to the contribution of this element in root water uptake. Silicon induced an increase in water potential through the osmoregulatory action of proline^13,14^. Allied to this, the activity of aquaporins was also likely to have increased. Aquaporins are proteins specialized in water uptake, which may have the expression of their genes activated by Si^15,70^. Thus, it can be stated that plants that received Si were further protected from tissue dehydration by two associated mechanisms: decreased water loss and increased water uptake by the mechanisms discussed above.

Silicon-induced maintenance of cellular hydration decreased photosynthetic damage in plants under water deficit (Fig. 4l) due to stomatal and nonstomatal changes. Stomatal changes were evidenced by the decrease in Gs (Fig. 4b, d) and E (Fig. 4e, g), possibly by the polymerization of part of the silica on the leaf surface^11^. However, Ci (Fig. 4f, h) increased despite the decrease in Gs, suggesting the involvement of nonstomatal factors in photosynthetic activation^19^.

Nonstomatal factors correlate with biochemical adjustment in the prevention and reduction of oxidative damage in plants. Prevention was evidenced by the maintenance of water status, which avoided losses in the photochemical efficiency of PSII. This was demonstrated by the highest values ​​of Fv/Fm assessed in the morning (Fig. 2i, k) and at the time of high temperature (Fig. 2j, l) for both species. This increase indicates that Si decreases damage to PSII reaction centers as most of the solar radiation was being used in the photochemical phase of photosynthesis^19^, preventing excessive ROS formation^56^.

Increased photochemical efficiency may be due to the higher content of Chl *a* (Fig. 3a, b), Chl *b* (Fig. 3c, d), and carotenoids (Fig. 3e, f), which maintain photosynthetic activity and prevent exposure of chloroplasts at high excitation energy levels.^57^ Other authors reported the effect of Si in increasing carotenoids, which are powerful antioxidants that decrease degradation of different organic compounds in the plant, including chlorophyll^61^.

Silicon plays an important role in protecting and increasing chlorophyll in plants. This favors light absorption by leaves, consequently increasing photosynthetic activity and the content of soluble sugars^71^ that may even compose cell walls. Silicon complexation with cell wall macromolecules occurs via sugar stabilization, similarly to the borate-mediated formose reaction^72^. The transfer of light energy from carotenoids to chlorophylls thus increases, favoring the protection of plants against stress^56^. Along with this, the effects of Si can also be due to increased dissipation of ROS formed from the activation of the enzymatic (Fig. 6a–f) and nonenzymatic (Fig. 5e-h) antioxidant defense system of plants.

Superoxidase dismutase activity in Si-nourished plants increased in both water regimes in sugarcane and in the water deficit regime in energy cane (Fig. 6a, b), demonstrating that Si collaborates in active oxygen inactivation by catalyzing O_2_^-^ to H_2_O_2_ and O_2_.^60^ Previous reports indicate higher levels of SOD activity as a mechanism that increases drought tolerance in sugarcane plants under water deficit conditions^73^. Therefore, Si would increase the tolerance of plants under severe water deficit because it stimulates SOD activity. In this sense, sugarcane is more efficient in the use of this biological mechanism as it can obtain this response even under adequate water conditions (Fig. 6a, b). However, despite the formation of H_2_O_2_, its content decreased due to the concomitant increase in the activity of the enzymes CAT (Fig. 6c, d) and APX (Fig. 6e, f) that use it as a substrate for the formation of H_2_O and O_2_^60^. These results agree with those of other authors^16^ who reported that Si application strengthened the antioxidant defense system and decreased lipid peroxidation and oxidative damage in sugarcane plants under water deficit stress.

In addition to activating the enzymatic antioxidant system, Si also provided a beneficial effect on the performance of nonenzymatic antioxidant agents such as proline (Fig. 5g, h) and phenolic compounds (Fig. 5e, f). The increased concentration of proline contributed to maintain cellular osmotic adjustment, increasing cell water potential by stimulating an increase in cellular homeostasis^14^. Proline can also act as a molecular chaperone, protecting the integrity of proteins and maintaining or increasing the activity of enzymes signaling ROS dissipation^74^. Thus, the increase in proline content improved the defense system of plants of both species fertilized with Si. The contents of phenolic compounds were also comparatively higher in sugarcane and energy cane plants that received Si supply (Fig. 4e, f). This increase collaborated with the decrease in ROS accumulation^63^. In other studies, the increase in phenolic content correlated with the decrease in ROS production in maize plants under water deficit^61^.

The physiological and biochemical mechanisms modified by Si in increasing the tolerance of plants grown under water deficit increased the WUE of plants. This is an important physiological attribute, especially for plants under drought conditions^75^. In this study, the water deficit decreased WUE, which in turn was improved by Si supply (Fig. 4i, k). The literature also indicates an increase of WUE by Si in sugarcane plants under water stress, but with the application of the element only via leaves^19^.

The beneficial effect of Si on the biological mechanisms under study attenuated severe water deficit damage on plants. Its application favored plant growth while decreasing leaf senescence, *i*.*e*., percentage of dry leaves (Fig. 7c, d). Moreover, silicon application increased the number of green leaves (Fig. 7a, b) and consequently leaf area (Fig. 7g, h), also increasing tillering (Fig. 7e, f) and dry matter production of leaves (Fig. 8a, b), culms (Fig. 8c, d), and shoots (Fig. 8e, f). Thus, the second hypothesis of this study was accepted, indicating that the enrichment of plants with Si applied in the seedling formation phase with complementation after transplanting provides a beneficial effect that attenuates water deficit damage up to initial culm formation. This occurred due both to the Si-induced mechanism that changes physiological parameters favoring the maintenance of cell hydration and to the biochemical benefits from the increased activity of enzymatic and nonenzymatic antioxidants, decreasing oxidative stress.

It is noteworthy that the benefit of Si in increasing the tolerance of plants under water restriction occurred in a relatively long period, from 7 to 160 days after transplanting, including the phase of high relative growth rate of the crop. In other words, it indicates the efficiency of production of new dry matter over the existing one, which occurs close to 84 to 135 days after transplanting.^7^ Reduction of stress in this stage of high dry matter accumulation rate may even have a beneficial impact on the next phenological stages of the crop. When Si attenuates water deficit, it favors tillering, which is one of the main components of productivity of sugarcane or energy cane. The more the plant tillers in the initial growth stage, the greater the productivity of the crop.

This research also made it clear that the use of Si under the conditions of this study can enable suboptimal irrigation for the cultivation of these species. This could save irrigation water denoting expressive environmental gains as it is a finite natural resource.

Further research should be conducted under field conditions so that the use of Si can be expanded for sustainable cultivation of sugarcane and energy cane crops in different production cycles and in drought areas.

## Conclusion

Silicon supply via fertigation in association with foliar spraying in the seedling formation phase with complementation after transplanting is efficient in increasing Si accumulation in the plants. The presence of Si in the plant attenuates severe water deficit damage up to initial culm formation, maintaining water and physiological balance by favoring the antioxidant defense system in sugarcane and energy cane plants.

The present research suggests Si supply in the seedling formation phase and after transplanting to the soil as a complementary strategy in areas with water restriction for the initial formation of culms of sugarcane and energy cane, especially in sandy soils and soils with low Si availability.

## Supplementary Information

Supplementary Information 1.

Supplementary Information 2.
