# Supplementary material for: Action of silicon on the activity of antioxidant enzymes and on physiological mechanisms mitigates water deficit in sugarcane and energy cane plants
Source: Sci Rep. 2022 Oct 19;12:17487. doi: 10.1038/s41598-022-21680-9 (PMC9581957; doi:10.1038/s41598-022-21680-9)
Supplement: Supplementary file 1 — Supplementary Information 1. [file 41598_2022_21680_MOESM1_ESM.docx]

**Action of silicon on the activity of antioxidant enzymes and on physiological** **mechanisms mitigates water deficit in sugarcane and energy cane plants**

Gelza Carliane Marques Teixeira^a^*, Renato de Mello Prado^a^, Antonio Márcio Souza Rocha^b^, Antonio Santana Batista de Oliveira Filho^a^; Gilmar da Silveira Sousa Junior^c^, Priscila Lupino Gratão^c^

^a^Laboratory of Plant Nutrition, Department of Soils and Fertilizers, São Paulo State University (UNESP), Jaboticabal, São Paulo, Brazil.

^b^Laboratory of Biogeochemistry, Department of Technology, São Paulo State University (UNESP), Jaboticabal, São Paulo, Brazil.

^c^Laboratory of Plant Physiology, Department of Biology Applied to Agriculture, São Paulo State University (UNESP), Jaboticabal, São Paulo, Brazil.

***Corresponding author:** gelzacarliane@hotmail.com (GCMT). Telephone: +551632097404. ORCID: 0000-0002-8062-482X

**Supplementary Figure**


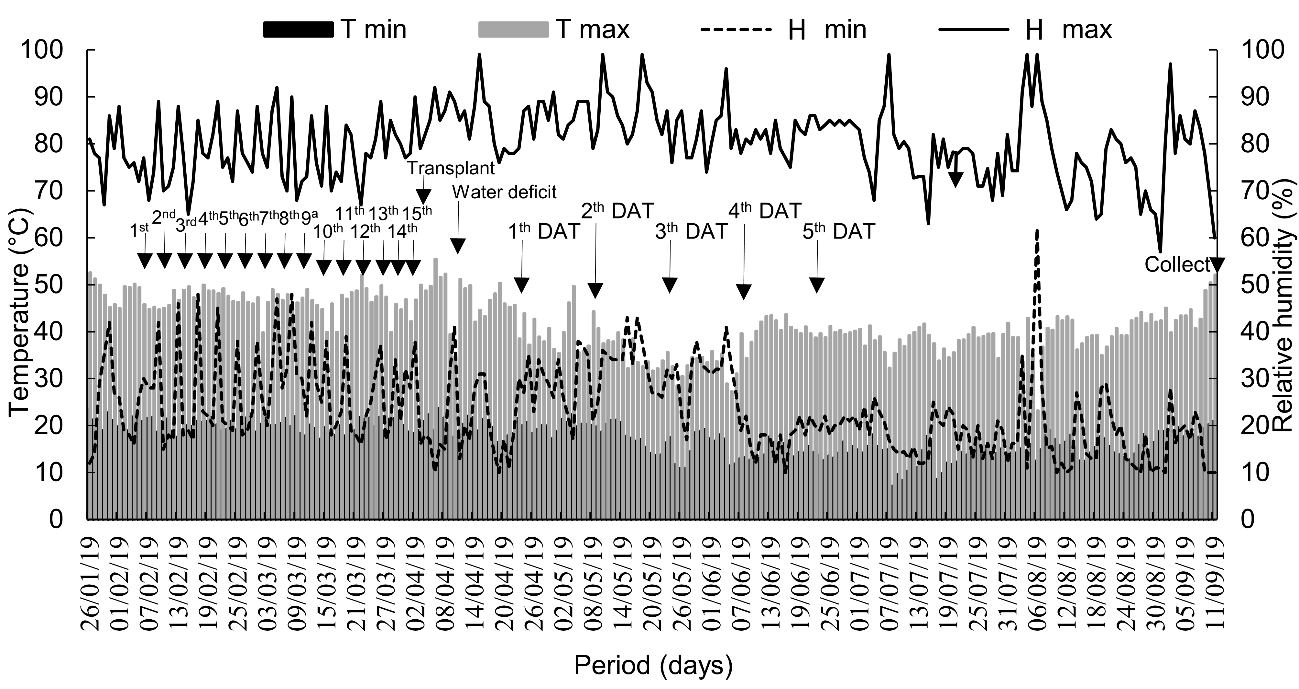


**Figure** **S1.** Maximum (T max) and minimum (T min) temperature and maximum (H max) and minimum (H min) relative air humidity in the greenhouse during the experimental period. Arrows indicate the dates of silicon application. DAT = days after transplanting.
